# Risk of MERS importation and onward transmission: a systematic review and analysis of cases reported to WHO

**DOI:** 10.1186/s12879-016-1787-5

**Published:** 2016-08-25

**Authors:** Chiara Poletto, Pierre-Yves Boëlle, Vittoria Colizza

**Affiliations:** 1 Sorbonne Universités, UPMC Univ Paris 06, INSERM, Institut Pierre Louis d’épidémiologie et de Santé Publique (IPLESP UMRS 1136), 75012 Paris, France; 2 Institute for Scientific Interchange Foundation, via Alassio 11/c, 10126 Torino, Italy

**Keywords:** MERS, Epidemic preparedness, Imported cases, Secondary transmission

## Abstract

**Background:**

The continuing circulation of MERS in the Middle East makes the international dissemination of the disease a permanent threat. To inform risk assessment, we investigated the spatiotemporal pattern of MERS global dissemination and looked for factors explaining the heterogeneity observed in transmission events following importation.

**Methods:**

We reviewed imported MERS cases worldwide up to July 2015. We modelled importations in time based on air travel combined with incidence in Middle East. We used the detailed history of MERS case management after importation (time to hospitalization and isolation, number of hospitals visited,…) in logistic regression to identify risk factors for secondary transmission. We assessed changes in time to hospitalization and isolation in relation to collective and public health attention to the epidemic, measured by three indicators (Google Trends, ProMED-mail, Disease Outbreak News).

**Results:**

Modelled importation events were found to reproduce both the temporal and geographical structure of those observed – the Pearson correlation coefficient between predicted and observed monthly time series was large (*r* = 0.78, *p* < 10^−4^). The risk of secondary transmission following importation increased with the time to case isolation or death (OR = 1.7 *p* = 0.04) and more precisely with the duration of hospitalization (OR = 1.7, *p* = 0.02). The average daily number of secondary cases was 0.02 [0.0,0.12] in the community and 0.20 [0.03,9.0] in the hospital. Time from hospitalisation to isolation decreased in periods of high public health attention (2.33 ± 0.34 vs. 6.44 ± 0.97 days during baseline attention).

**Conclusions:**

Countries at risk of importation should focus their resources on strict infection control measures for the management of potential cases in healthcare settings and on prompt MERS cases identification. Individual and collective awareness are key to substantially improve such preparedness.

**Electronic supplementary material:**

The online version of this article (doi:10.1186/s12879-016-1787-5) contains supplementary material, which is available to authorized users.

## Background

Concern with the Middle East respiratory syndrome (MERS) remains high among public health authorities worldwide due to the failure to stop the spread of the disease three years after its first description [1]. As of February 2016, 26 countries reported cases, for a total of 1,626 laboratory-confirmed cases of infection with MERS [2]. Cases outside the Middle East region were either travellers or had close contact with imported cases.

The majority of case importation events led to little or no secondary transmission, as expected due to the low human-to-human transmission risk [3–7]. Yet, in May 2015, a case imported to South Korea triggered the largest outbreak outside the Middle East, with a total of 186 confirmed cases [2]. This large epidemic was quite unexpected and calls for a better understanding of the potential for MERS dissemination worldwide and risk of onward transmission.

It has been proposed that countries having a high risk of MERS importation were those receiving the most air passengers from the Middle East [5, 8]. Joint analysis of incidence data in the Middle East region with international travel flows allowed estimating the expected number of MERS introductions in countries outside the affected area [9, 10], for example on coming back from pilgrimage in Mecca [9] or distinguishing between residents and visitors [10]. In the latter, predictions were based on cumulative attack rates per country in the affected area [10], thus disregarding the strong temporal nature of MERS epidemic [11].

In case of importation of a case, experience has shown that household and nosocomial transmission was possible [12–14] with infrequent large outbreaks as in South Korea [2]. A marked heterogeneity in the epidemic outcome following importation has been observed and characterized by transmission trees in South Korea [15] and theoretical results regarding the potential for multiple generations [16] and role of super-spreading events [16, 17]. Yet a comprehensive understanding of the risk factors for local transmission once a MERS case is imported is still missing.

Here we aimed at providing a comprehensive analysis of the risk of MERS importation and subsequent onward transmission through a systematic analysis of all known MERS importation events. We produced predictions based on air traffic data for the risk of MERS importation and estimated the expected number of symptomatic cases imported in countries outside the affected area – here defined as Saudi Arabia, Qatar, Oman, Kuwait, Jordan, United Arab Emirates, Yemen, Bahrain, and also referred to as Middle East. The analysis accounted for seasonal variations of air traffic flows and for temporal and spatial variations of MERS incidence in the Middle East. Predictions were validated against confirmed importations and lack thereof. Focusing on onward transmission, we analysed the risk for local transmission according to sources of heterogeneity in terms of individual awareness (time to hospital admission, declaration of history of travel), country’s reaction (baseline hospital protocols, heightening of infection control strategies), cultural aspects and local customs (e.g. health-seeking behaviour) and phase of the outbreak. We modelled the outcome of importation events according to how cases were identified and managed to provide quantitative estimates on the expected number of secondary cases in the community and in the hospital setting. We finally explored whether MERS awareness in different communities at the time of importation affected case management.

## Methods

### Data collection on imported cases

Confirmed cases by World Health Organisation (WHO) between September 1, 2012 and July 31, 2015 were considered with the exclusion of repatriations events [18]. We focused on cases with clear and documented travel origin. We thus excluded cases with no association to travel and cases in countries in the Middle East region, here defined as Saudi Arabia, Qatar, Oman, Kuwait, Jordan, United Arab Emirates, Yemen, Bahrain, where continuous sporadic cases are documented.

For each importation event, we collected from the scientific literature, Disease Outbreak News of WHO (DON-WHO), and other official public health sources the following information: dates of travel and symptoms onset, date of suspected MERS-CoV infection and date of confirmation, date of hospital admission, date of case isolation or death, declaration of travel history, number of secondary cases generated by the imported case, hospitalization history. Information was primarily extracted by one of us (CP) and double-checked by others (PYB, VC).

### Model-predicted number of imported cases

We modeled imported cases out of Middle East during the same period as above based on air-traffic data and observed incidence in the source region. We used air-traffic worldwide provided by the International Air Transport Association [19]. The dataset refers to 2013 and includes monthly number of origin–destination trips connecting 3362 urban areas distributed on all countries. We extended the dataset to the whole study period by assuming perfect annual periodicity. For reported cases in Middle East we considered the official counts published in the Disease Outbreak News of WHO and recovered from [20, 21]. We aggregated incidence by week and we grouped cases spatially in 20 regions corresponding to the provinces of Saudi Arabia and the other countries. We computed the number of MERS-CoV infections exported each week to a destination country outside Middle East assuming that infected individuals have the possibility to travel before hospitalized. We accounted for the distribution of time from infection to hospitalization, computed as the convolution of the incubation period distribution and that of onset to hospitalization (see Additional file 1 for the details). To estimate actual number of importations we adjusted incidence in Middle East for reporting ratio. The latter was computed by assuming 100 % detection accuracy in imported cases in European and North-American countries, where alertness and surveillance have been high to detect importations [22–24].

Eventually, we computed the predictive probability of the weekly number of importation cases worldwide depending on how many cases were reported in the Middle East in the past month. Further details are reported in the Additional file 1.

### Risk of transmission following importation

We studied the factors affecting the risk of transmission following importation. We used bias reduced logistic regression to analyse the outcome of each imported case, classified as “with secondary case” vs. “without secondary case” [25].

We then modelled the number of secondary cases following importation assuming a Poisson process. Several versions of the model were explored to test potential determinants of the observed heterogeneity in transmission. The first factor considered was the presence/absence of dependence of transmission on the setting (community vs. hospital), as accounted for by models S+ and S- respectively. We then considered possible dependence on the total duration of the transmission risk period (with, model D+, vs. without, model D-). This was defined as the duration from symptoms onset or date of travel, if travelling after symptoms onset, to isolation or death. For models S+ we used setting-specific transmission risk periods. Eventually we compare presence/absence of heterogeneity in transmission between patients (with, model P+, vs. without, model P-). The decomposition tested in the models accounts for the aspects that were shown to be relevant in the heterogeneity of transmission by the risk analysis. Notice that in all models tested we assumed that no transmission is possible after patient isolation. To account for duration we set the mean of the Poisson distribution to μ_C_d_C_ + μ_H_d_H_, where d_C_ and d_H_ were the number of days in the community and in hospital, and μ_C_ and μ_H_ the average number of secondary cases per day in each setting. Overdispersion in transmission was introduced by replacing μ_H_ with a Gamma-distributed random parameter m_H_ with mean μ_H_ and standard deviation σ_H_. We further distinguished between a model with over-dispersion of transmission only in the hospital (P+/D+/S+), and a model accounting for such a level of heterogeneity in both hospital and community (P++/D+/S+). All listed transmission hypotheses yielded 8 different models that were fitted to the data and compared by the Akaike Information Criterion (AIC). More details on the analysis are reported in the Additional file 1.

### Collective attention and awareness and relation with imported case history

As possible external factor affecting the history of importation events, we studied the effect of attention or awareness as obtained from various digital sources. We focused on three indicators: the popularity of the search query [“novel coronavirus” OR “MERS-CoV”] in Google Trends, as an indicator for collective attention in the general public; the number of alerts published by ProMED-mail with the same keywords, as an indicator of attention in the international infectious disease community; and the number of DON on MERS published by WHO, as an indicator of official source of information for public health authorities.

Each source provided a time-series spanning the whole study period; values were scaled to range between 0 and 1 at the time-series maximum. For each indicator, we computed the correlation coefficient between the time spent in the community or in the hospital and the attention level at the occurrence date of these events. We defined periods of high attention as those where the indicator value was in its upper quartile distribution. We compared the length of the periods spent in the community or in the hospital before isolation occurring in high attention periods or in the remaining baseline periods*.* Alternative definitions of the threshold for high attention periods were tested (Additional file 1).

## Results

### Risk of MERS importation

**Fig. 1:**
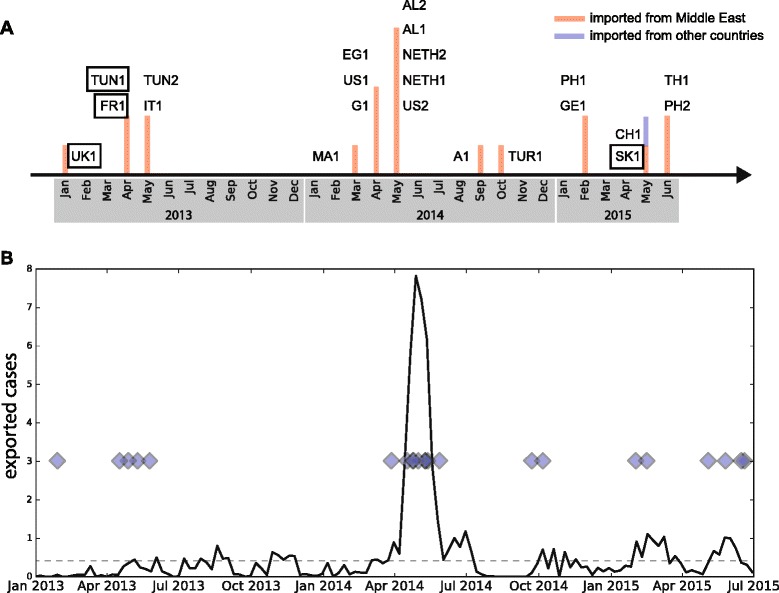
MERS importation events. **a** Timeline of confirmed importation events. The height of the bar is proportional to the number of importations registered in the month, and imported cases’ IDs are listed close to the bar. Importation events causing secondary transmissions are highlighted with a box. All cases originated from travels from the Middle East (red bars), except the case imported in China (CH1, blue bar) who travelled from South Korea. **b** Expected weekly number of MERS exportation to countries out of Middle East as a function of time. The average weekly number of exportations is indicated with the dashed line. Blue diamonds indicate observed exportations (detailed in panel **a**)

A total of 22 importation events were reported worldwide (see Table 1 and Additional file 1): nine in Europe (41 %), six in Asia (27 %), five in Africa (23 %), and two in North America (9 %). All cases were symptomatic. Confirmed secondary cases following importation were observed on four occasions (two in Europe, one in Africa, and one in Asia). Two further secondary cases in Italy were not confirmed by WHO [18, 26, 27] and were only investigated in the sensitivity analysis. Exception made for the CH1 case who travelled from South Korea, all cases were originating from Middle East. Importation events were reported throughout the whole study period, with half of them occurring during 2014. Cases with associated confirmed transmissions following importation occurred during 2013 (UK1, FR1, TUN1) and 2015 (SK1). Timeline of importations is summarised in Fig. 1a.

**Table 1:** Case importation events of MERS to countries outside of the Middle East region

#	Case ID	Country	Date of travel	Date of onset of symptoms	Date of MERS confirmation	Sec. cases
1	UK1	United Kingdom	28/1/13	24/1/13	8/2/13	2
2	FR1	France	17/4/13	22/4/13	7/5/13	1
3	IT1	Italy	25/5/13	24/5/13	31/5/13	0
4	TUN1	Tunisia	28/4/13	28/4/13	8/5/13	1
5	TUN2	Tunisia	10/5/13	11/5/13	16/5/13	0
6	MA1	Malaysia	28/3/14	04/4/14	14/4/14	0
7	G1	Greece	17/4/14	prior to traveling	18/4/15	0
8	EG1	Egypt	25/4/14	22/4/14	26/4/14	0
9	US1	United States	24/4/14	18/4/14	2/5/14	0
10	US2	United States	1/5/14	1/5/14	9/5/14	0
11	NETH1	The Netherlands	10/5/14	01/5/14	13/5/14	0
12	NETH2	The Netherlands	10/5/14	05/5/14	14/5/14	0
13	AL1	Algeria	28/5/14	23/5/14	30/5/14	0
14	AL2	Algeria	29/5/14	23/5/14	30/5/14	0
15	A1	Austria	22/9/14	prior to traveling	29/9/14	0
16	TUR1	Turkey	6/10/14	25/9/14	-	0
17	PH1	Philippines	1/2/15	26/1/15	10/2/15	0
18	GE1	Germany	8/2/15	11/2/15	7/3/15	0
19	SK1	South Korea	4/5/15	11/5/15	20/5/15	31
20	CH1	China	26/5/15	21/5/15	28/5/15	0
21	TH1	Thailand	15/6/15	10/6/15	18/6/15	0
22	PH2	Philippines	19/6/15	30/6/15	4/7/15	0

The comparison between predicted and observed exported cases from Middle East is reported in Fig. 1b. Confirmed MERS importations occurred when computed expected number of cases was large. The Pearson correlation coefficient between the observed and predicted number of cases flying out of the Middle-East region each month was 0.78 (*p* < 10^− 4^).

**Fig. 2:**
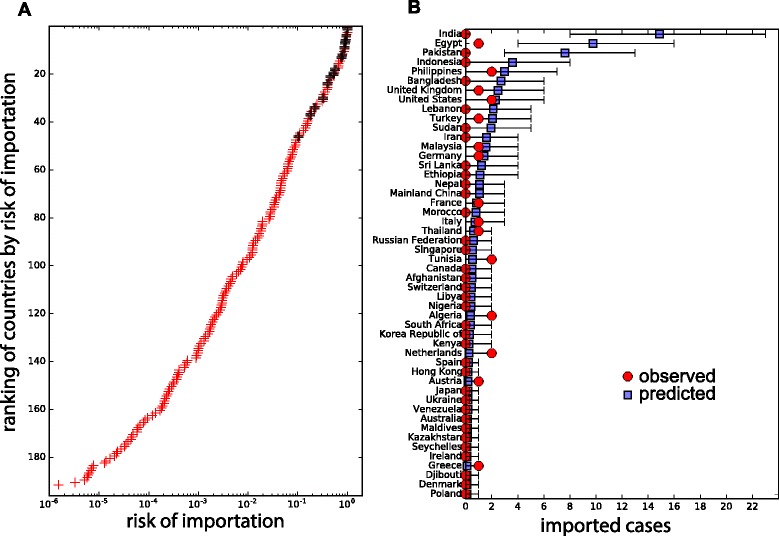
MERS importations by country. **a** Risk of importation by country worldwide. Confirmed importations are signalled by black symbols. **b** Observed vs. predicted number of MERS imported cases by country over the whole study period. Top 50 countries ranked by the predicted number of importations are shown (including all reported importations). Bars indicate the 95 % prediction interval

At the country level (Fig. 2a), we found that countries experiencing importation cases had indeed a higher expected probability of importing a case (Wilcoxon test *p* < 10^− 4^). Five of the ten countries with the highest probability of importing cases actually reported importation cases: these were mostly European countries. The remaining five did not report importation cases despite being at high risk of importation: these included countries in South-East Asia (e.g. India, Pakistan, Indonesia and Bangladesh). Observed number of importation cases matched prediction in all countries, except for India, Egypt and Pakistan (Fig. 2b). Among European and North-American countries observed importations were even more strongly associated with model predictions: 100 % of the top five countries registered at least one case, and 70 % of top ten, see Additional file 1: Figure S2.

**Fig. 3:**
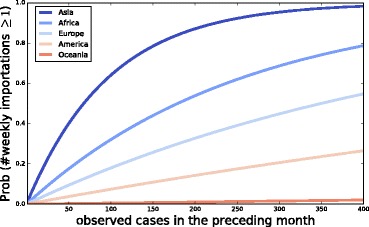
Probability of observing at least one case within a week as a function of the observed epidemic activity at the source in the preceding month. Different curves correspond to different continents

Building on the good agreement between observed and predicted cases worldwide, we analyse the risk of importation events that would be expected according to a given disease activity in the Middle East. Figure 3 reports the probability of observing at least one imported case in each continent according to the number of cases observed in the Middle East over the last month (see Additional file 1: Figure S3 for predicted number of importations). Following a month with increased disease incidence in the Middle-East (more than 100 cases – for example April 2014 or August 2015), the probability of having at least 1 case would be 64 % in Asia, 32 % in Africa, 18 % in Europe, 7 % in the Americas and 0 in Oceania.

### Risk of transmission following importation

**Fig. 4:**
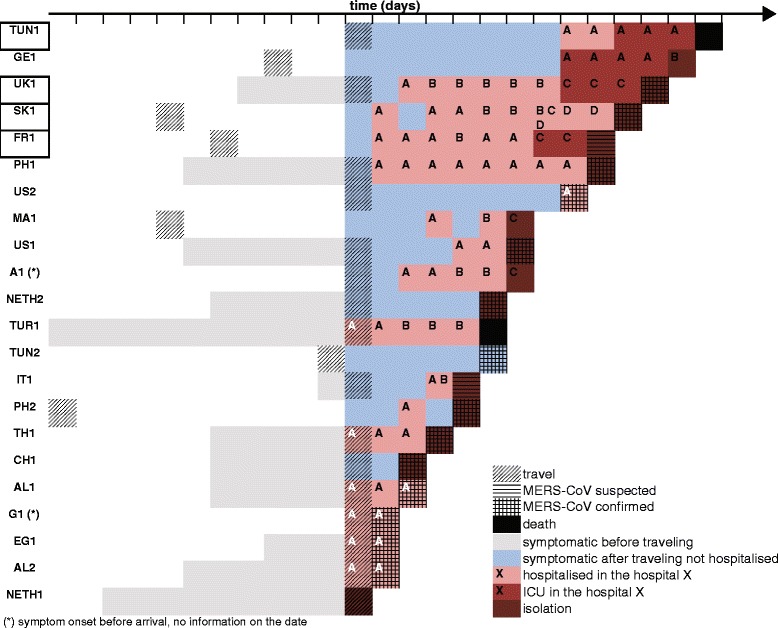
Case history of MERS importations. Cases are aligned to the beginning of the infectious risk period, i.e. the most recent between the importation date and the date of symptoms onset. Days in the infection risk period are color-coded according to the patient’s history, ending with isolation (*dark red*) or death (*black*). Importation events are sorted by duration of the infection risk period. Where no information was available on time of isolation, we assumed infection risk period to end with case confirmation. A box is used to highlight cases that led to secondary transmission

For each MERS-confirmed imported case, we reconstructed the detailed case history (Fig. 4). In the four events where local transmission was confirmed, the duration of the transmission risk period, from symptoms onset or date of travel if symptomatic to isolation or death, was longer than in others (11.8 days vs. 5.4, *p* = 0.007). FR1, SK1, and UK1 were characterised by a long hospitalisation period (ten days for UK1, nine days for FR1 and SK1) and a short period in the community (two days or less). TUN1 showed a longer period in the community (eight days) than in the hospital (six days). Importation events with no local transmission had shorter duration of hospitalisation on average (2.9 vs. 8.5 days). The number of visited hospitals prior to isolation ranged between one and four (four visited by SK1).

Results of the bias reduced logistic regression show that imported cases with the longest transmission risk period more frequently had secondary cases (OR = 1.7, CI 95 % [1.2,6.7]), all the more when more time was spent in the hospital (OR = 1.7 [1.2,7.3]). Number of clinics visited was also associated to increased risk of transmission. The small sample made it difficult to conclude for other characteristics, although it suggested an increased probability for secondary cases if onset occurred after travelling, and if history of travel to the Middle East region was not reported (Table 2).

**Table 2:** Risk factors for secondary cases after importation (univariate analysis)

Variable	OR	95 % CI	P
Onset before importation	0.2	[0.00, 1.7]	0.19
Infection risk period (per day)	1.7	[1.2, 6.7]	0.04
Time before hospitalization (per day)	1.1	[0.7, 1.6]	0.62
Time in the hospital (per day)	1.7	[1.2, 7.3]	0.02
Number of visited healthcare facilities	3.3	[1.2, 25.4]	0.05
Declared history of travel^a^ (No vs. Yes)	8.2	[0.32, 625]	0.21

^a^information available for 15 cases out of 22

Results of the AIC comparison among transmission models are reported in Table 3. We found that the models allowing over-dispersion of transmission in the hospital provided a better fit to the data. The best fit was obtained with model P+/D+/S+. Model P+/D+/S-, where transmission in and outside the hospital was similar, performed nearly as well as the best model. Model P++/D+/S+, where overdispersion was present in the community and in the hospital performed more poorly, essentially because the variance of the random coefficients was not well estimated. Community parameters were estimated with a very large variance showing that the likelihood was almost flat and the model difficult to identify.

**Table 3:** AIC values for all model tested

Model			AIC
P	D	S	
-	-	-	197.1
-	+	-	150.0
-	+	+	141.1
+	-	-	45.5
+	+	-	43.4
+	+	+	43.3
++	+	+	46.0

**Fig. 5:**
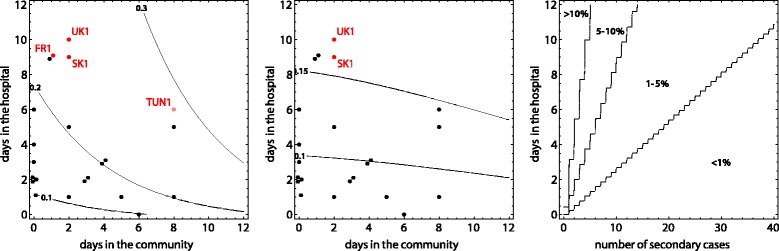
Model-predicted probability of secondary cases after importation. (*left*, *middle*) Model predicted probability of at least one secondary case (left), of more than one secondary case (middle) as a function of the time spent in the community and in the hospital prior to isolation or death. In red countries that experienced local transmission generating at least one secondary case (*left*) or more than one secondary case (*middle*). (*right*) Model predicted probability of the number of secondary cases as a function of the time spent in the hospital, assuming 3 days in the community before hospitalisation

Parameters estimated with the two best fitting models are listed in Table 4. In the best fitting model estimated average daily secondary cases was ten times smaller in the community than in the hospital. The importation events causing secondary transmissions had larger model-predicted probabilities of transmission (Fig. 5). Finally, the model showed that most importations were likely to cause less than five secondary cases, especially when time to isolation was short. It also showed that outbreaks leading to more than 30 cases were possible, although with a relatively small probability (1–5 % predicted for the South Korean episode). In model P+/D+/S-, the probabilities of a large number of secondary cases following importation were larger than in the best fitting model (see Additional file 1: Figure S4).

**Table 4:** Parameters estimated for the two best fitting models

Model			Parameters			
P	D	S	μ_C_	μ_H_	σ_C_	σ_H_
+	+	-	0.15 [0.04,1.69]		0.53 [0.16, 8.7]	
+	+	+	0.02 [0.0, 0.12]	0.20 [0.03, 9.0]	-	0.73 [0.20, 75.0]

Accounting for the unconfirmed secondary cases in Italy yielded similar association between infectious risk period and increased transmission risk, but less marked evidence for setting-specific differences in transmission (Additional file 1).

### Collective attention and awareness and relation with imported case history

**Fig. 6:**
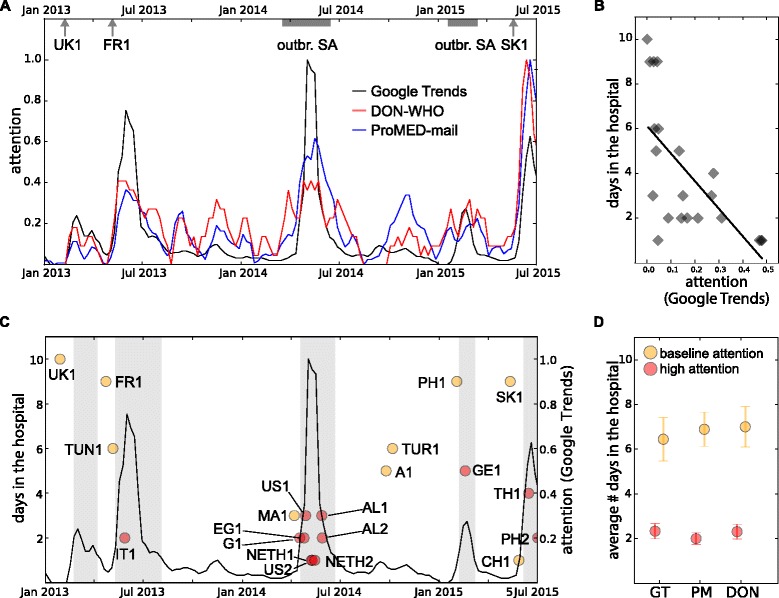
Relation between attention and time from hospitalisation to isolation. **a** Attention as measured by Google Trends, ProMED-mail and DON-WHO from January 2013 to July 2015. Dates corresponding to specific epidemic events are shown on the top of the plot. **b** Duration of hospitalisation versus attention (from the Google Trends indicator) at the time of admission to the hospital. **c** Timeline of attention (Google Trends, right axis) and duration from hospitalisation to isolation for each imported case (left axis), at the time of admission to the hospital. Periods of high attention are indicated by vertical shaded areas. Importation events are coloured according to attention level at their time of hospitalisation (red for high attention, orange otherwise). **d** Average duration from hospitalisation to isolation in periods of high and baseline attention for three indicators (GT= Google Trends, DON= Don of WHO, PM= ProMED-mail). Error bars show standard errors

Trends of attention measured by the three indicators showed similar profiles, with periods of high popularity interspersed within periods of lower attention in the various sources (Fig. 6a). The correlations between indicators were large, ranging between 0.65 (Google Trends vs. DON-WHO, *p* < 10^− 4^) and 0.86 (ProMED-mail vs. DON-WHO, *p* < 10^− 4^). The latter is expected as ProMED-mail contains all news of DON-WHO. ProMED-mail and DON-WHO showed more variation over time than Google Trends, where fewer and more distinct peaks were observed. Peaks were more likely to occur following MERS related events (Fig. 6a), such as e.g. the confirmation of MERS infection in imported cases worldwide (UK1, FR1) or the outbreaks in Saudi Arabia (Spring 2014 and February 2015) [2].

We compared the length of the periods spent in the community or in the hospital before isolation with the attention level at the occurrence date of these events. The duration from hospitalisation to isolation was larger in periods of low attention for all three indicators. The correlation coefficient was -0.66 (*p* = 0.001) between duration and Google Trends attention level (Fig. 6b), -0.69 (*p* = 0.0005) for ProMED-mail, and -0.58 (*p* = 0.005) for DON-WHO (Additional file 1: Figure S5).

More precisely, we found that the imported cases in Italy (IT1), Greece (G1), United States (US1 and US2), Algeria (AL1 and AL2), Egypt (EG1), the Netherlands (NETH1 and NETH2), Germany (GE1), Thailand (TH1), and Philippines (PH2), all hospitalised during periods of high attention (Fig. 6c), had average time from hospitalisation to isolation of 2.33 ± 0.34 days compared to 6.44 ± 0.97 days computed for cases occurring when attention was lower (Fig. 6d).

All indicators of attention yielded similar results, with more rapid isolation in periods of high attention (Fig. 6d). Two cases occurring outside Google Trends high attention periods were nevertheless isolated quickly and captured by the other indicators (CH1 occurring in a period of high attention for ProMED-mail and DON-WHO, MA1 during high attention for DON-WHO). There was no substantial difference when criteria for the calculation of the moving average and thresholds defining high attention were varied (Additional file 1).

Attention was not found to impact duration of the community period. For the case of attention measured by Google Trends, for example, we obtained a Pearson correlation coefficient between attention and length of stay in the community equal to 0.22 (*p* = 0.32). Average values in periods of high and baseline attention were respectively 2.85 ± 0.83 and 2.44 ± 0.74. Also in this case results were robust in varying criteria for the moving average calculation and the high attention definition.

## Discussion

The large number of cases reported in the South Korean outbreak following a MERS case importation generated substantial concern on the risks that countries face towards importation and possible onward transmission. Our quantitative analysis provide important information that can help preparedness and response to such events: MERS international dissemination is strongly associated to air travel flows combined with cases incidence in the source area; transmission is more likely to occur in hospitals; large outbreaks are possible though rare; and high attention to MERS epidemic in the public and among professionals lead to efficient case management.

The role of air travel as driver for pathogen international dissemination has been considered in several prospective studies [28–37], with few retrospective validations [33, 38–42]. For the case of MERS [5, 8–10], qualitative comparisons between predictions and observed importations were presented [9, 10]. Our analysis produces the first quantitative assessment of the accuracy of model predictions for the risk of importation worldwide accounting for all countries with and without reported importations. In addition, importation risk and expected number of imported cases is evaluated across time. The temporal factor is often neglected when dealing with subcritical epidemic as in the case of MERS and overall attack rates are generally considered [10]. MERS epidemic was shown however to display a strong temporal component, both in the zoonotic and human-to-human transmission [11]. Here we found indeed that such temporal variation also affects the expected number of exportations from the Middle East region. The largest concentration of exportations (nine episodes from March to May 2014) occurred during the outbreaks affecting the provinces of Riyadh and Makkah in Saudi Arabia in Spring 2014, causing a 18-fold increase in the expected number of exportations. Spatial resolution is also important, as already highlighted in [10] with a country-level description. Here we considered a total of 20 regions in the affected area, including the countries of Saudi Arabia, Qatar, Oman, Kuwait, Jordan, United Arab Emirates, Yemen, Bahrain similarly to [10], and additionally disaggregating Saudi Arabia into its 13 provinces [11].

A further element of novelty of the present study is its ability to provide projections on the expected number of importations by country with no prior knowledge on either MERS epidemiology or under-reporting ratio. This is possible by relying on three main assumptions: (i) 100 % detection accuracy of MERS surveillance in countries of European Union and North America; (ii) uniform under-reporting of cases in the affected area in time and space [11]; (iii) homogeneous mixing between travellers and local residents.

The third assumption may be responsible for some of the limitations of the model. The major discrepancy we found between predictions and observations is the large risk of importation predicted for India, Pakistan, and Indonesia, though these countries did not report any MERS case. This was also observed in [10]. It may suggest that cases were imported in these countries but went undetected. However it may also be due to non-homogeneity in the mixing or travel behaviour of classes of individuals, namely travellers to the affected area vs. region residents, that is known to impact the conditions for pathogen dissemination [43]. Non-homogeneous mixing may affect travellers’ probability to be infected by the virus because of altered risk of contact with zoonotic sources or of exposure to nosocomial outbreaks. This may vary among travellers and be related for instance to different purpose of visit (e.g. short-visit tourists vs. seasonal workers). The similarity of findings regarding high-risk countries between our study and previous work [10], notwithstanding different modelling assumptions, suggests that country-specific aspects may be determinant for the observed discrepancy. On one side there is a surveillance system whose accuracy may vary by country. On the other, purpose of visit and associated mixing may be country-related. India, Pakistan, and Indonesia are indeed in the top 4 nationalities in the expat community in Saudi Arabia [44].

The detailed analysis of the history following importation provided important findings on the risk of a local outbreak generated by an infected traveller. While no secondary cases were reported in the majority of observed importation events, four events led to one (in two instances), two, or 31 secondary cases. This heterogeneity was captured by our models through large overdispersion in transmission, in agreement with previous works [15–17]. Our findings suggest that the probability to observe a future MERS importation leading to a number of secondary cases larger than the South Korean one is of the order of 1-5 %, consistent with prior work on the full nosocomial South Korean outbreak [15]. In line with another study, the risk of observing a secondary case is predicted to be larger than 20 % if isolation in hospital is not fast (>one week), independently of the time spent in the community [16].

The observed variability in epidemic outcomes may result from at least four characteristics. First, the duration of the transmission risk period in the destination country, from date of importation or symptoms onset to isolation or death, is clearly an important risk factor for local transmission. The longer the transmission risk period, the larger are the opportunities for susceptible individuals to be exposed to the infectious case, both in the community and after hospitalization.

Second, nosocomial transmission of MERS appeared more efficient than transmission in the community. This points to the need for focusing interventions on rapid case identification and effective isolation and for improving infection control protocols in hospitals to prevent transmission. The large outbreak observed in South Korea was indeed attributed to sub-optimal infection prevention and control measures in hospitals [45]. These findings are in line with previous analyses on MERS transmission in healthcare settings and with modelling studies evaluating the impact of mechanisms to control SARS spread in 2003 [15, 46–48].

The third aspect pertains to heterogeneity in case finding and management. For example, imported cases could visit one to four health-care facilities before getting a diagnosis. This behaviour may be based on individuals’ decisions but it may also be induced by country-specific regulations of the national health-care system that determines how patients can access professionals or it may be influenced by local customs. This behaviour was found to be associated with a higher probability of secondary transmission in our study, indicating that the local practice of seeking care in multiple health-care facilities may have contributed to the initial spread in South Korea, in agreement with the findings of Ref. [45].

The fourth aspect is represented by lack of individual awareness regarding the potential risk that was found to be a contributing factor increasing transmission risk. Indeed, not reporting a history of travel to the health practitioners increased the probability of having secondary cases (although not statistically significant). For emerging diseases whose non-specific symptoms preclude a fast diagnosis, travel history is a critical element for patient assessment. Moreover, cases who experienced onset of symptoms before importation were less likely to generate secondary cases. Traveling while ill from a country where a MERS outbreak is ongoing increased individual awareness on the infection risk and induced a more precautionary individual behaviour. Clearly, these two aspects are correlated, as 70 % of the cases who declared history of travel also travelled while ill.

We explored three digital indicators for collective attention and awareness corresponding to different communities: general population, professionals and health authorities. Digital sources have been recently used in the context of early detection and surveillance [49, 50]. They have also been used as a source to study public concern in response to an ongoing epidemic threat [49, 51].

Our analysis showed that general public’s attention measured by Google Trends is highly correlated with the amount of news circulating amongst the international infectious disease community (ProMED-mail) and of official reports published by the WHO. Its curve in time shows the presence of high peaks followed by a quick decline. This is a common behaviour generally found in the social media response following specific triggering events [49, 52, 53]. In our study, events triggering collective attention can be identified with unexpected episodes of case importations or resurgence of the epidemic in the affected area.

Here we found that MERS confirmation and isolation during peaks of collective attention were considerably faster. This suggests that an increased collective awareness may induce changes in the management of the patient allowing the health system to successfully reduce the time from admission to isolation. Being this time critical for transmission risk, increased awareness is identified as a key factor to control the generation of cases after MERS importation. Similar results were also obtained for the indicators measuring awareness in the public health and infectious diseases community. The case importation in South Korea occurred when attention was at its baseline level (as measured by all indicators), and indeed it was reported that MERS appearance was “unfamiliar to most physicians” in the country [54].

No correlation was found instead between collective attention and time to admission. This suggests that collective attention may preferably act on the case management by health authorities, whereas individual awareness may more likely be responsible for individual change of health-seeking behaviour, as discussed previously.

The impact of collective awareness and public health mobilization strategies in response to an epidemic has already been observed in past outbreak. As SARS outbreak progressed, reduced duration from onset to hospital admission and reduced number of hospital visits per patient were observed due to overall increased awareness and public health recommendations [47, 55–57]. Similarly, during the South Korean outbreak of MERS, infection control measures strengthened, and the delay from illness onset to confirmation shortened as the epidemic progressed [58, 59].

There are however two important differences between our findings and the above. First, awareness and concerns are known to be stronger for the population experiencing the outbreak (as in the above cases) than for yet unaffected populations (as in our study) [53]. Nonetheless, our findings show that higher awareness, even when induced by non-local events, allow countries to better manage an importation episode. Second, here we were able to explicitly measure awareness in time and relate it to a quantifiable change of behaviour (case management), further illustrating its impact on transmission. From the use of surveys to digital data, quantifying awareness has become an important aspect of epidemic surveillance and control [49, 51, 60–62]. Few studies have however measured how variation in awareness affects an epidemic during its course [50, 63]. Moreover, MERS poses additional challenges as public attention is known to wane rapidly in response to external events [64] unless it is prompted by additional triggering events or mounting concern, as it happened with the rapidly increasing number of cases of Ebola epidemic [5]. The subcritical nature of MERS epidemic, with an incidence characterized by subcritical spread and sporadic peaks [11], induces a similarly fragmented timeline of attention. For example, the large peak of Spring 2014 increased the risk of MERS dissemination. The management of imported cases during that period however benefited from an increased attention to the disease epidemic, and no secondary transmission was observed. Conversely, despite the risk of importation was much lower during the beginning of 2012 and Spring 2015, the few importations observed during that period were able to initiate local transmission and threaten the health security of the destination countries. By analysing the various aspects characterizing the epidemic, our analysis is able to identify the factors that may help improving the reaction of countries at risk of importation.

Other limitations of our study need to be mentioned. We assumed that no importation events had been missed in Europe and estimated under-reporting in the Middle-East accordingly. However, if 1 importation case had been missed in Europe, underreporting would be 25 % rather than 18 %, and the model-predicted number of cases worldwide increase by 10 % over the period. If one further assumes that secondary transmission was unlikely for these unrecognized cases, the probability of transmission following importation would be overestimated. For example, taking the model-predicted number of importation cases worldwide for the period as exact, the probability of local transmission would have been 6 % (4 among 70) rather than the current 18 % (4 among 22).

Furthermore, the second best-fitting model among the ones considered for transmission following importation, with only marginally larger AIC, did not support setting-specific transmission. Our main result is however in line with previous findings on nosocomial transmission of MERS in the Middle East [15]. In addition, there were two secondary cases in the Italian episode that were not confirmed by official sources. Including these two cases in the analysis, we still found that longer time to isolation was associated with more secondary transmission, although evidence on setting-specific differences in transmission was less marked (see Additional file 1).

## Conclusions

We evaluated the risk of MERS exportation worldwide integrating seasonal air traffic flows and time-varying incidence of cases in the affected area, and accounting for under-reporting of cases. We conducted a comprehensive analysis of all reported MERS imported cases to validate modelled importation risk and assess onward transmission. Our findings confirm the critical role of air travel in the risk of international dissemination. In case of MERS introduction, prompt identification of the infection in patients seeking medical attention, strict infection control measures, and effective isolation of the patient in the nosocomial setting are key to efficient prevention and control of the outbreak. Increasing awareness at collective and public health levels worldwide is found to be associated with higher local preparedness, prompter and strengthened precautionary measures and isolation procedures to prevent further spread. Our findings provide a quantitative assessment for public heath authorities to face the variability associated to importation risk and potential for local transmission, to inform preparedness plans, and identify the critical measures that should be considered to reduce the likelihood of future outbreaks.

## Additional files

Additional file 1: The file contains detailed information on importation event history, detailed explanation of the statistical analysis, sensitivity analysis. (PDF 6580 kb) Additional file 2: The file contains timeline of attention from the three digital sources considered in the paper. (XLSX 40 kb)
