# Supplementary material for: Risk of MERS importation and onward transmission: a systematic review and analysis of cases reported to WHO
Source: BMC Infect Dis. 2016 Aug 25;16(1):448. doi: 10.1186/s12879-016-1787-5 (PMC5000488; doi:10.1186/s12879-016-1787-5)
Supplement: Additional file 1: — The file contains detailed information on importation event history, detailed explanation of the statistical analysis, sensitivity analysis. (PDF 6580 kb) [file 12879_2016_1787_MOESM1_ESM.pdf]

Additional file 1: Risk of MERS importation and onward transmission: a systematic review and analysis of cases reported to WHO

Chiara Poletto[a] PhD, Pierre-Yves Boëlle[a] PhD, Vittoria Colizza[a,b] PhD

[a]Sorbonne Universités, UPMC Univ Paris 06, INSERM, Institut Pierre Louis d'épidémiologie et de Santé Publique (IPLESP UMRS 1136), AP-HP, Hôpital Saint-Antoine, F75012, 27 rue Chaligny, 75012 Paris, France.

[b]Institute for Scientific Interchange Foundation, via Alassio 11/c, 10126 Torino, Italy.

Table of Contents

Methods 2
Model-predicted number of imported cases 2
Predicting future risk of importation 3
Risk of transmission following importation 3
Describing secondary cases after importation 3
Model formulations 3
Predicted probabilities 4
Collective attention and awareness and relation with imported case history 4
Results 6
Detailed data of MERS-CoV importation events 6
Risk of MERS importation 7
Risk of transmission following importation 8
Predictions for the second best fitting model 8
Sensitivity analysis: accounting for probable transmissions reported in Italy 9
Collective attention and awareness and relation with imported case history 10
References 11

Methods

Model-predicted number of imported cases

We computed the number $e(t, d)$ of MERS-CoV infections exported during week t to destination country d outside the Middle-East region as:

$$e(t,d) = \sum_{c} f(t,c,d)\, W_{IH} W_{IO}^{-1}\ o(t,c)/pop(c)\,/\,\rho$$

where $o(t,c)$ was the frequency of MERS-CoV cases with onset in week t in region c with population $pop(c)$, $f(t,c,d)$ was the number of passengers flying from region c to country d on week t, W_{IH} and W_{IO} were matrices described below and ρ was the ratio of reporting (i.e. reported cases to actual cases) in the Middle East. Summation was over 20 source regions in the Middle East.

We first reconstructed the incidence curves $i(t,c)$ for each Middle East region. For some cases, the date of onset was missing and was imputed using date of hospitalization or date of report (1,2). We averaged all results over 20 imputed time series. The variability due to imputation was however very small in the results (<1%). Then, the number of cases with onset in week t, $o(t,c)$, was linked to the incidence of infection in the preceding weeks using the incubation period distribution $w_{IO}(k)$ (i.e. the fraction of infected cases with disease onset between $k-1$ and k weeks) according to:

$$\begin{pmatrix} o(1) \\ o(2) \\ \vdots \\ o(T) \end{pmatrix} = W_{IO} \cdot \begin{pmatrix} i(1) \\ i(2) \\ \vdots \\ i(T) \end{pmatrix}, \text{ where } W_{IO} = \begin{pmatrix} w_{IO}(1) & 0 & \cdots & 0 \\ w_{IO}(2) & w_{IO}(1) & \ddots & \vdots \\ \vdots & \ddots & \ddots & 0 \\ 0 & 0 & \ddots & w_{IO}(1) \end{pmatrix}$$

For example, we have $o(2) = i(1)w_{IO}(2) + i(2)\,w_{IO}(1)$, i.e. cases with onset in week 2 are those infected in week 1 with 2 weeks incubation period and cases infected in week 2 with 1 week incubation period. We took W_{IO} from a study of South Korean Outbreak (3) (average 6.7 days – Figure S1). The corresponding values of W_{IO} were ($w_{IO}(1) = 0.6$, $w_{IO}(2) = 0.38$, $w_{IO}(3) = 0.02$).

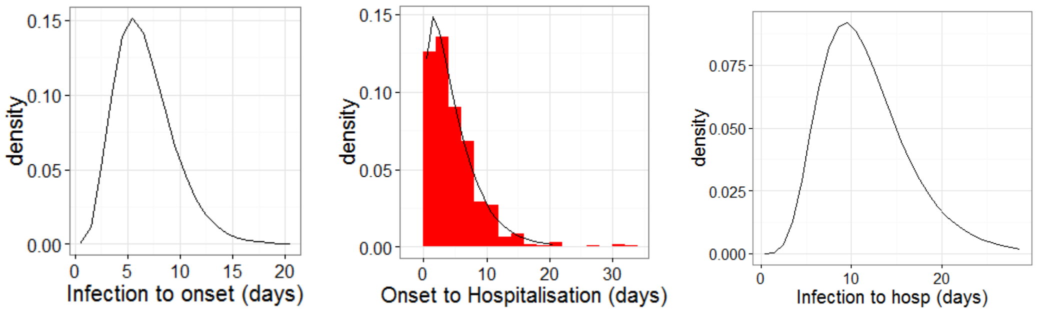

Figure S1: Distributions of Incubation period (infection to onset), onset to hospitalization, and infection to hospitalization for MERS-CoV infection.

We then hypothesized that infected cases could travel outside the Middle-East area until hospitalization. Indeed, cases may travel before onset of the disease, but also after onset, as illustrated by 10 of the 22 exportation cases (45%) who travelled when already ill. Time from infection to hospitalization can be split in two parts: infection to onset, i.e. incubation, and onset to hospitalization. The distribution can then be computed as the convolution of the incubation period distribution and that of onset to hospitalization (W_{IH}). For onset to hospitalization, we analyzed 521 cases from the Middle East reported to WHO (out of 1291) for whom both onset and hospitalization dates were available. Onset to hospitalization took 4.4 days on average, so that the average duration from infection to hospitalization was 11.1 days (Figure S1). We obtained $w_{IH}(1) = 0.16$, $w_{IH}(2) = 0.57$, $w_{IH}(3) = 0.22$, $w_{IH}(4) = 0.05$ for the portion of cases hospitalized the 1st, 2nd, 3rd and 4th week after infection. From the distribution we computed the prevalence of infected cases not already hospitalized in week t $p(t)$ by:

$$p(t) = \left(1 - \frac{w_{IH}(1)}{2}\right) i(t) + \left(1 - w_{IH}(1) + \frac{w_{IH}(2)}{2}\right) i(t-1) + \left(1 - w_{IH}(1) + w_{IH}(2) - \frac{w_{IH}(3)}{2}\right) i(t-2) +$$

$(1\text{-}w_{IH}(1) + w_{IH}(2) - w_{IH}(3) + \frac{w_{IH}(4)}{2})i(t-3)$,

applying an actuarial survival method to take into account hospitalization (cases hospitalized in a given week only contributed half a week to exposure during this week). In matrix form this reads:

$$\begin{pmatrix} p(1) \\ p(2) \\ \vdots \\ p(T) \end{pmatrix} = W_{IH} \cdot \begin{pmatrix} i(1) \\ i(2) \\ \vdots \\ i(T) \end{pmatrix}, \text{ where } W_{IH} = \begin{pmatrix} 1 - w_{IH}(1)/2 & 0 & \cdots & 0 \\ 1 - w_{IH}(1) - w_{IH}(2)/2 & 1 - w_{IH}(1)/2 & \ddots & \vdots \\ \vdots \ \ddots & & \ddots & 0 \\ 0 \quad 0 & & \ddots & 1 - w_{IH}(1)/2 \end{pmatrix}$$

The latter combined with the matrix equation above yield the prevalence of cases in travelers from the series of number of disease onset with time.

Last, we calibrated ρ by setting the predicted number of imported cases in Europe and North America (United States and Canada) equal to the registered importations in these countries over the period (10 importations). Europe was defined as the 32 countries participating in the ECDC surveillance. We obtained ρ = 82% [47% - 164%] - confidence interval based on the likelihood ratio test.

Predicting future risk of importation

We computed the predictive probability of the weekly number of importation cases worldwide depending on how many cases were reported in the Middle East in the past month. For these computations, the number of air passengers to each destination was fixed at the annual average, disregarding seasonal variation.

We computed P(E|O), where E is the number of importation cases and O the number of reported cases in the Middle East.

The distributions of observed cases is $P(O = o) = \sum_x \binom{x}{o} \pi^o (1-\pi)^{x-o} p(x)$, where π is the probability of report, fixed here at 0.82 and p(x) the distribution of monthly incidence. We described p(x) as a Gamma distribution with mean 31 and standard deviation 40, to reflect typical values between 2012 and 2015.

The joint distribution of E and O is $P(E = k \,\&\, O = o) = \sum_x \binom{x}{o} \pi^o (1-\pi)^{x-o} \exp(-r\, x)\, (r\, x)^k / k!\, p(x)$. The coefficient r summarized the link between incident cases and exported cases. It was computed as $r = \sum_{d,c} \frac{f(c,d)\gamma_c}{pop_c} \alpha$, where *d* sums over all destination countries in a continent, *c* sums over provinces in the Middle East, *f(c,d)* is the average weekly number of air passengers from *c* to *d*, γ_c is the fraction of all cases occurring in province *c* and pop_c its population, and α the expected portion of cases from the last month not hospitalized. Values of r were 3.18e-3 for Africa, 6.34e-4 for the Americas, 8.43e-3 for Asia, 1.63e-3 for Europe and 4.27e-3 for Oceania.

Risk of transmission following importation

Describing secondary cases after importation

For a typical importation case, information is summarized as (n, d_C, d_H), where n is the number of secondary cases and d_X the number of days spent in setting X (Community or Hospital). We modelled n as a Poisson random variable with mean $E(n) = f(d_C, d_H)$, as described below.

Model formulations

Models included:

- dependence on time before isolation: We used $f(d_C, d_H) = m_C + m_H$(model D-) or $f(d_C, d_H) = m_C d_C + m_H d_H$ (model D+), where m_X corresponded to the average number of secondary cases before isolation (D-) or average number of secondary cases *per day* before isolation (D+).

- dependence on the setting: We assumed that the number of secondary cases in the community and the hospital were the same ($m_C = m_H$; model S-) or not ($m_C \neq m_H$; model S+).

- overdispersion: We accounted for over-dispersion by using random parameters. More precisely, we adopted gamma-distributed random patient-level parameters (m_C, m_H) with mean and standard deviation (μ_C, σ_C) and (μ_H, σ_H). Overdispersion could be present in the hospital, in the community or in both settings.

We studied the set of models obtained by combining these characteristics listed in Table S1. In all cases, parameters of interest were the (daily) number of secondary cases (μ) and, depending on the model, the standard deviation of the random parameters. Models were fit with Mathematica.

Table S1: List of models tested and summary of their characteristics.

Model				Secondary cases distribution	Hypotheses
**1**	**P**	**D**	**S**		
**2**	-	-	-	$n_i \sim Poisson(\mu)$	Same for all cases, Independent of duration
**3**	-	+	-	$n_i \sim Poisson(\mu(d_C+d_H))$	Same for all cases
**4**	-	+	+	$n_i \sim Poisson(\mu_H\, d_{H,i} + \mu_H\, d_{H,i})$	$\mu_C \neq \mu_H$; Same for all cases
**5**	+	-	-	$n_i \sim Poisson(m_i)$<br>$m_i \sim Gamma(\mu, \sigma)$	m : Random effect
**6**	+	+	-	$n_i \sim Poisson(m_i(d_{C,i}+d_{H,i}))$<br>$m_i \sim Gamma(\mu, \sigma)$	$m_{C,i} = m_{H,i} = m_i$
**7**	+	+	+	$n_i \sim Poisson(\mu_C\, d_{C,i} + m_{H,i}\, d_{H,i})$<br>$b_{H,i} \sim Gamma(\mu_H, \sigma_H)$	overdispersion only in the hospital
**8**	++	+	+	$n_i \sim Poisson(m_{C,i}\, d_{C,i} + m_{H,i}\, d_{H,i})$<br>$m_{X,i}, \sim Gamma(\mu_X, \sigma_X)$	overdispersion in community & hospital

Predicted probabilities

For the best-fitting model (model 7; P+/D+/S+), we computed the predicted probabilities of more than *k* secondary cases after importation as *E(P(n >= k))* for *k=0, 1* and *2*.

The probabilities were:

$$P(n \geq K | \mu_C, \alpha_H, \beta_H) = 1 - e^{-\mu_C d_C}(1 + d_H\beta_H)^{-\alpha H} \sum_{k=0}^{K} \sum_{j=0}^{k} \frac{(\mu_C d_C)^j}{j!} \left(\frac{d_H b_H}{1 + d_H b_H}\right)^{k-j} \frac{\Gamma(a_H + k - j)}{\Gamma(a_H)\,(k-j)!}$$

where $\alpha_H = (\mu_H/\sigma_H)^2$ and $\beta_H = \sigma_H^2 / \mu_H$ are the shape and scale of the gamma distribution.

Collective attention and awareness and relation with imported case history

For each of the three indicators, being $a(t)$ the attention during the week preceding the date *t*, we built the time-series formed by the moving average $\tilde{a}(t) = \frac{1}{T}\sum_{s=0}^{s<T} a(t-s)$, with *T* explored between 1 and 4. A time-series of duration of hospitalization $d_{H,i}(t_i)$ was built by associating for each patient *i* the number of days of hospitalization $d_{H,i}$ to the date of hospitalization $t_{H,i}$. Analogously, a time-series of duration of the period in the community $d_{C,i}(t_i)$ is built by associating for each patient *i* the number of days in the community $d_{C,i}$ to the latest between the dates of arrival and symptoms onset $t_{C,i}$. In order to compare $d_{H,i}(t_{H,i})$ and $d_{C,i}(t_{C,i})$ with $\tilde{a}(t)$ we computed the linear interpolation of $\tilde{a}(t)$ to build a daily time-series $\tilde{a}_{daily}(t)$ and then we extracted the sub-series $\tilde{a}_{daily}(t_{X,i})$ (with $X = H, C$) formed by the values of the daily attention time-series corresponding to the dates of hospitalization and arrival/symptoms onset for each case. The correlation between $d_{H,i}$ and $\tilde{a}_{daily}$ is then measured by the Pearson correlation coefficient. Results reported in the main paper are obtained with *T=3*.

In order to quantify the impact of attention on the duration of hospitalization and period in the community we identified periods of high attention and compare average length of stay in the community/hospital conditioned to high and baseline attention, $AV[d_{X,i}]_{HA}$ and $AV[d_{X,i}]_{BA}$ (with $X = H, C$). Periods of high attention are defined as

the ones for which $\tilde{a}(t) > \tilde{a}_{TH}$, with $\tilde{a}_{TH}$ equal to the 75% percentile of $\tilde{a}(t)$. We tested alternative definitions of high attention periods by considering 60% and 90% percentile, $\tilde{a}_{TH} = AV[\tilde{a}(t)] + STD[\tilde{a}(t)]$, with $AV[\tilde{a}(t)]$ average and $STD[\tilde{a}(t)]$ standard deviation of the whole time-series $\tilde{a}(t)$, and by considering also an annually varying threshold obtained by computing the 75% percentile for each year separately.

Results

Detailed data of MERS-CoV importation events

Table S2: Detailed Information on imported cases.

Case ID	Country	Date of travel	Declared history of travel	Date of onset of symptoms	Date of MERS confirm. (§)	Hospitalization history (¶)(***)	Sec. cases	Source
**UK1**	United Kingdom	28/1/13	-	24/1/13	8/2/13	30/1/13 A (daily visit)<br>31/1/13 B<br>5/2/13 C (ICU)<br>8/2/13 C (isolation)	2	(4)
**FR1**	France	17/4/13	Y	22/4/13	7/5/13<br>suspect.<br>1/5/13	23/4/13 A<br>26/4/13 B (daily visit)<br>29/4/13 C (ICU)<br>1/5/13 C (isolation)	1	(5)
**IT1**	Italy	25/5/13	Y	24/5/13	31/5/13<br>suspect.<br>29/5/13	28/5/13 A<br>28/5/13 B<br>29/5/13 B (isolation)	0	(6),(7)
**TUN1**	Tunisia	28/4/13	-	28/4/13	8/5/13	06/5/13 A<br>08/5/13 A (ICU)<br>10/5/13 deceased	1	(8)
**TUN2**	Tunisia	10/5/13	Y	11/5/13	16/5/13	No hospit.	0	(8)
**MA1**	Malaysia	28/3/14	Y	04/4/14	14/4/14	7/4/14 A (daily visit)<br>9/4/14 B<br>10/4/14 C (Isolation)	0	(9)
**G1**	Greece	17/4/14	Y	prior to traveling	18/4/15	17/4/14 A (†)	0	(10)’(11)
**EG1**	Egypt	25/4/14	-	22/4/14	26/4/14	25/4/14 A (†)	0	(12)
**US1**	United States	24/4/14	Y	18/4/14	2/5/14<br>suspect.<br>1/5/14	28/4/14 A<br>1/5/14 A (isolation)	0	(13)
**US2**	United States	1/5/14	Y	1/5/14	9/5/14	9/5/14 A (†)	0	(14)’(15)
**NETH1**	The Netherlands	10/5/14	Y	01/5/14	13/5/14	10/5/14 A (isolation)	0	(16)
**NETH2**	The Netherlands	10/5/14	Y	05/5/14	14/5/14	15/5/14 A (isolation)	0	(16)
**AL1**	Algeria	28/5/14	Y	23/5/14	30/5/14	28/5/14 A (†)	0	(17)’(18)
**AL2**	Algeria	29/5/14	Y	23/5/14	30/5/14	29/5/14 A (†)	0	(17)(18)
**A1**	Austria	22/9/14	Y	prior to traveling	29/9/14	24/9/14 A<br>26/9/14 B<br>28/9/14 C (isolation)	0	(19)
**TUR1**	Turkey	6/10/14	Y	25/9/14	-	06/10/14 A<br>08/10/14 B<br>11/10/14 deceased	0	(20)
**PH1**	Philippines	1/2/15	-	26/1/15	10/2/15	2/2/15 A<br>10/2/15 B (isolation)	0	(21)
**GE1**	Germany	8/2/15	-	11/2/15	7/3/15	19/2/15 A (ICU)<br>23/2/15 B (isolation)	0	(22,23)
**SK1**	South Korea	4/5/15	N	11/5/15	20/5/15	12/5/15 A (daily visits)<br>14/5/15 A (daily visits)<br>15/5/15 A (daily visits)<br>15/5/15 B<br>17/5/15 C<br>17/5/15 D<br>20/5/15 E (isolation)	31 (*)	(24),(25),(3)
**CH1**	China	26/5/15	N **(**)**	21/5/15	28/5/15	28/5/15 A (isolation)	0	(26)
**TH1**	Thailand	15/6/15	-	10/6/15	18/6/15	5/6/15 A<br>8/6/15 B	0	(27)
**PH2**	Philippines	19/6/15	-	30/6/15	4/7/15	2/7/15 A (daily visit)<br>4/7/15 B (daily visit)<br>4/7/15 C (isolation)	0	(28)**,**(29)

(*) Number of secondary cases recovered from(24); we attributed as secondary a case in hospital B with unknown transmission route.
(**) Refers to lack of declaration of history of close contact with a confirmed MERS case during his stay in South Korea.
(***) When no notification of hospital transfer was reported we assumed that no transfer occurred.
(§) Date in which MERS-CoV infection was suspected is also reported when available and different from the date of MERS-CoV confirmation.
(¶) Daily visits to clinics and general practitioners are counted as hospitals.
(†) No date of isolation available, date of confirmation is taken instead.

Risk of MERS importation

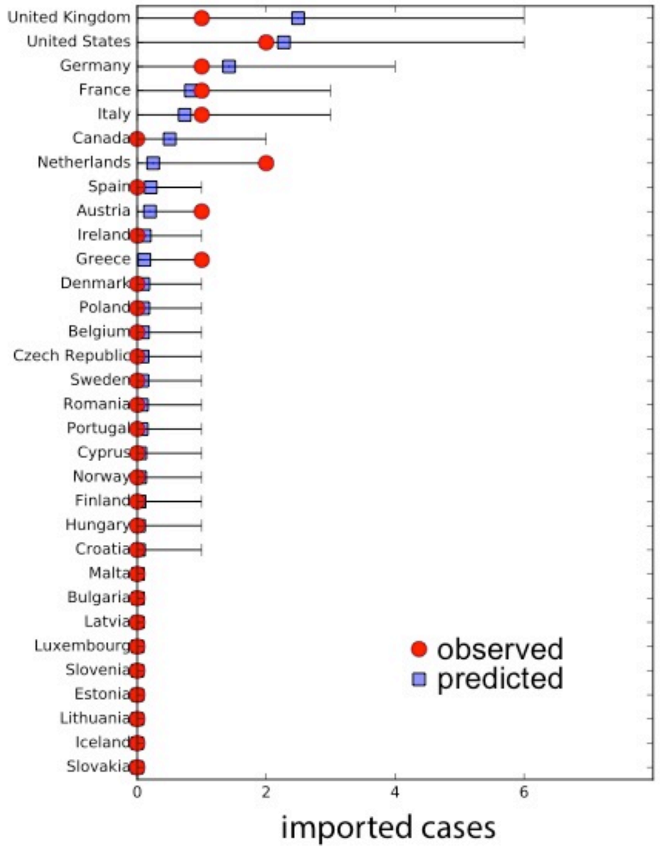

Figure S2: Predicted vs. observed number of cases in Europe and North America. Number of cases are integrated over the whole study period. Bars indicate the 95% prediction interval.

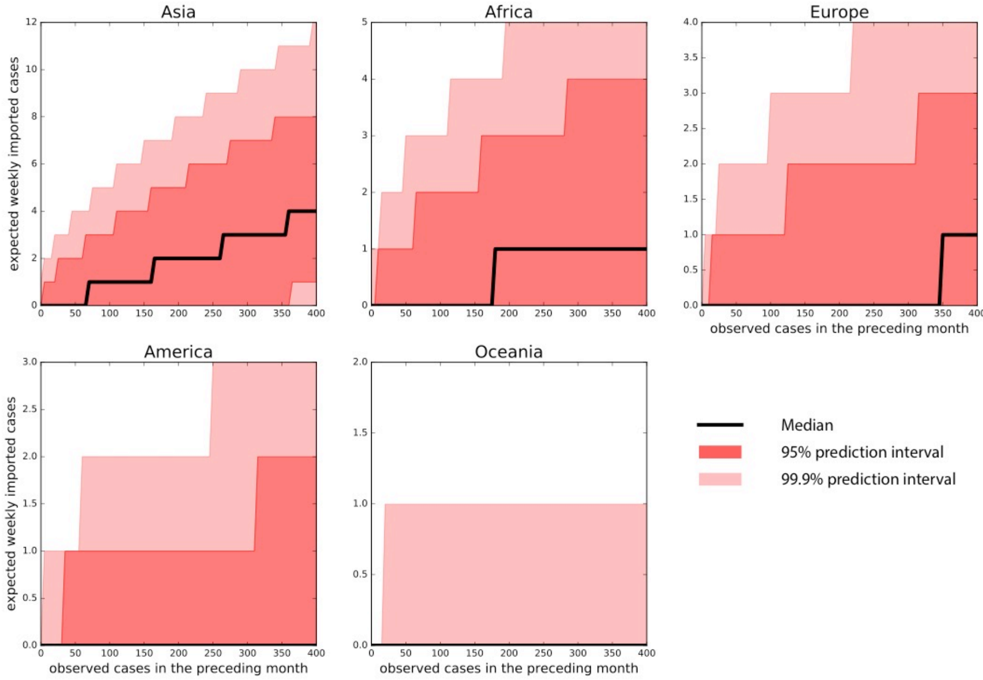

Figure S3: Expected number of cases within a week as a function of the observed epidemic activity at the source in the preceding month. Different panels correspond to different continents. Shaded areas indicate 95% and 99.9% prediction intervals.

Risk of transmission following importation

Predictions for the second best fitting model

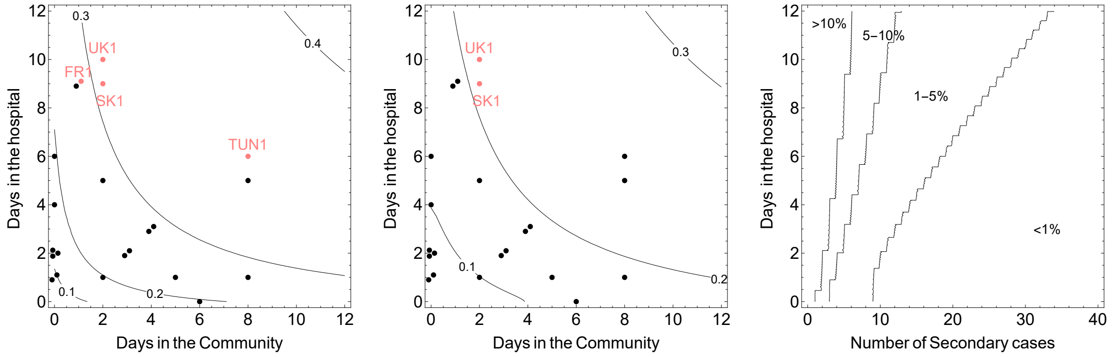

Figure S4: Predicted probabilities of at least one secondary case (left) and two or more (middle) for model P+/D+/S- (continuous lines). Black and red dots indicate the combinations of lengths of stay in hospital and community for the 22 cases analyzed. Red dots correspond to the importation events that caused at least one case on the left, and at least two cases on the right. (right) Model predicted probabilities of outbreak sizes according to time in the hospital before isolation.

Sensitivity analysis: accounting for probable transmissions reported in Italy

In the Italian importation event, there was uncertainty regarding 2 secondary transmission cases occurring in the community. These 2 cases were finally not confirmed by WHO and were not considered in the main analysis. Here, these 2 cases were included.

Table S3: Risk factors for secondary cases after importation (univariate analysis) for the probable transmissions reported in Italy

Variable	OR	95%CI	P
**Onset before importation**	0·4	[0·04, 2·8]	0·37
**Time to isolation (per day)**	1.4	[1·1, 2.6]	0·05
**Time before hospitalization (per day)**	1·1	[0·7, 1·6]	0·62
**Time in the hospital (per day)**	1·5	[1·1, 3.0]	0·02
**Number of visited healthcare facilities**	3·2	[1·2, 17.8]	0·04
**Declared history of travel[*] (No vs. Yes)**	4·6	[0.2, 190]	0·34

Results of logistic regression are reported in Table S3. We did not observe any substantial difference with the baseline scenario. Imported cases with the longest infection risk period more frequently had secondary cases. Duration of hospitalisation and number of clinics visited were associated to increase risk in transmission as well.

Table S4: AIC values for all model tested Scenario for the probable transmissions reported in Italy.

**Model**			**AIC**
**P**	**D**	**S**	
-	-	-	196·5
-	+	-	151·2
-	+	+	143·2
+	-	-	52·3
+	+	-	49·8
+	+	+	49·9

As above, models including overdispersion provided a better fit (Table S4). The best model was in this case P+/D+/S-, with only a minor difference in AIC with respect to P+/D+/S+. Differences in parameters values with respect to the baseline scenario are very small (Table S5).

Table S5: Parameters estimated for the two best fitting models. Scenario for the probable transmissions reported in Italy.

**Model**			**Parameters**			
**P**	**D**	**S**	μ_C	μ_H	σ_C	σ_H
+	+	-		0·17 [0·05, 1·14]		0·51 [0·17, 4·6]

+	+	+	0·02[0·0, 0·15]	0·25[0·04, 2·9]	-	0·73 [0·23, 20]

Besides logistic regression showed the important role of nosocomial transmission, heterogeneity in transmission was not found by AIC selection criterion to be a critical ingredient for modeling secondary case generation. We conclude then that accounting for the two Italian secondary cases led to a less marked evidence for setting-specific transmission.

Collective attention and awareness and relation with imported case history

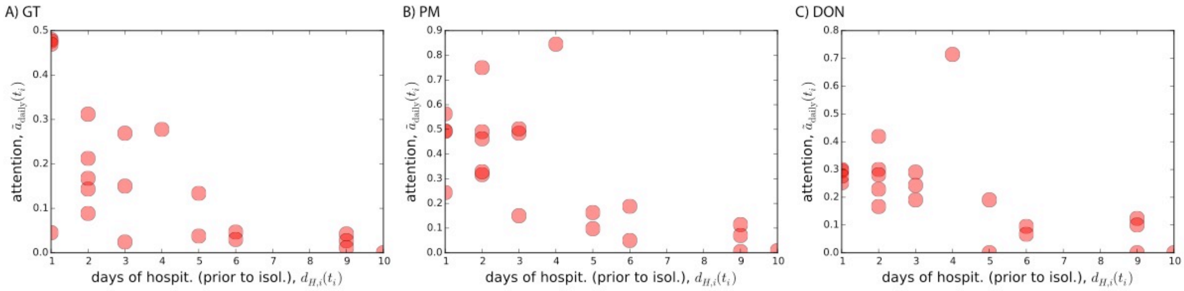

Figure S5: Scatter plots of attention $\tilde{a}_{\text{daily}}(t_i)$ versus duration of hospitalisation $d_{\text{H,i}}(t_i)$ for Google Trends (GT), ProMED-mail (PM) and DON-WHO (DON). *T=3.*

Table S6: Pearson correlation coefficient between $d_{\text{H,i}}(t_i)$ and $\tilde{a}_{\text{daily}}(t_{H,i})$. Comparison between different values of *T*.

T	**Google Trends**	**ProMED-mail**	**DON-WHO**
**1**	-0·59 (p= 0·004)	-0·75 ($p<10^{-4}$)	-0·70 (p= 0·0004)
**2**	-0·62 (p= 0·003)	-0·71 (p= 0·0003)	-0·59 (p= 0·005)
**3**	-0·66 (p= 0·001)	-0·69 (p= 0·0005)	-0·58 (p= 0·005)
**4**	-0·68 (p= 0.0007)	-0·69 (p= 0·0005)	-0·62 (p= 0·003)

Table S7: Duration of hospitalization, conditioned to high vs. baseline attention for different values of *T*. Average and standard error with threshold measured from the 75% percentile of the whole attention time-series.

	**Google Trends**		**ProMED-mail**		**DON-WHO**	
T	$AV[d_{\text{H,i}}(t_i)]_{HA}$ ± ***SEM***	$AV[d_{\text{H,i}}(t_i)]_{BA}$ ± ***SEM***	$AV[d_{\text{H,i}}(t_i)]_{HA}$ ± ***SEM***	$AV[d_{\text{H,i}}(t_i)]_{BA}$ ± ***SEM***	$AV[d_{\text{H,i}}(t_i)]_{HA}$ ± ***SEM***	$AV[d_{\text{H,i}}(t_i)]_{BA}$ ± ***SEM***
**1**	2·93 ± 0·56	7·00 ± 1·03	2·31 ± 0·38	7·00 ± 0·85	2·27 ± 0·45	6·10 ± 0·88
**2**	2·33 ± 0·34	6·44 ± 0·97	2·31 ± 0·38	7·00 ± 0·85	2·18 ± 0·38	6·20 ± 0·88
**3**	2·33 ± 0·34	6·44 ± 0·97	2·00 ± 0·26	6·88 ± 0·76	2·31 ± 0·33	7·00 ± 0·90
**4**	2·33 ± 0·34	6·44 ± 0·97	2·00 ± 0·26	6·88 ± 0·76	2·08 ± 0·25	7·37 ± 0·68

Table S8: Duration of hospitalization, conditioned to high vs. baseline attention for different definitions of the high attention threshold $\tilde{a}_{TH}$. Average and standard error with *T=3.* Average and standard error with *T=3.*

60%PERC = 60% percentile; 90%PERC = 90% percentile; 75%PERC$_y$ = 75% percentile for each year separately; AV+$STD = AV[\tilde{a}(t)] + STD[\tilde{a}(t)]$.

	Google Trends		ProMED-mail		DON-WHO	
$\tilde{a}_{TH}$	$AV[d_{H,i}(t_i)]_{HA} \pm SEM$	$AV[d_{H,i}(t_i)]_{BA} \pm SEM$	$AV[d_{H,i}(t_i)]_{HA} \pm SEM$	$AV[d_{H,i}(t_i)]_{BA} \pm SEM$	$AV[d_{H,i}(t_i)]_{HA} \pm SEM$	$AV[d_{H,i}(t_i)]_{BA} \pm SEM$
**75%PERC**	2·33 ± 0·34	6·44 ± 0·97	2·00 ± 0·26	6·88 ± 0·76	2·31 ± 0·33	7·00 ± 0·90
**60%PERC**	3·06 ± 0·54	7·40 ± 1·15	2·53 ± 0·37	8·00 ± 0·74	2·26 ± 0·31	7·71 ± 0·69
**90%PERC**	2·00 ± 0·40	5·14 ± 0·80	2·11 ± 0·33	5·58 ± 0·87	2·00 ± 0·35	5·38 ± 0·82
**75%PERC$_y$**	2·33 ± 0·34	6·44 ± 0·97	2·09 ± 0·27	6·3 ± 0·88	2·18 ± 0·28	6·2 ± 0·92
$\boldsymbol{AV + STD}$	2·00 ± 0·35	5·38 ± 0·82	2·11 ± 0·33	5·58 ± 0·87	2·00 ± 0·35	5·38 ± 0·82

References

1. Chowell G, Blumberg S, Simonsen L, Miller MA, Viboud C. Synthesizing data and models for the spread of MERS-CoV, 2013: key role of index cases and hospital transmission. Epidemics. 2014 Dec;9:40–51.

2. Poletto C, Colizza V, Boëlle P-Y. Quantifying spatiotemporal heterogeneity of MERS-CoV transmission in the Middle East region: A combined modelling approach. Epidemics. 2016 Jun;15:1–9.

3. Cowling B, Park M, Fang V, Wu P, Leung G, Wu J. Preliminary epidemiological assessment of MERS-CoV outbreak in South Korea, May to June 2015. Eurosurveillance. 2015 Jun 25;20(25):21163.

4. Health Protection Agency (HPA) UK Novel Coronavirus Investigation team. Evidence of person-to-person transmission within a family cluster of novel coronavirus infections, United Kingdom, February 2013. Euro Surveill Bull Eur Sur Mal Transm Eur Commun Dis Bull. 2013;18(11):20427.

5. Mailles A, Blanckaert K, Chaud P, van der Werf S, Lina B, Caro V, et al. First cases of Middle East Respiratory Syndrome Coronavirus (MERS-CoV) infections in France, investigations and implications for the prevention of human-to-human transmission, France, May 2013. Euro Surveill Bull Eur Sur Mal Transm Eur Commun Dis Bull. 2013;18(24).

6. Puzelli S, Azzi A, Santini M, Di Martino A, Facchini M, Castrucci M, et al. Investigation of an imported case of Middle East Respiratory Syndrome Coronavirus (MERS-CoV) infection in Florence, Italy, May to June 2013. Eurosurveillance. 2013 Aug 22;18(34):20564.

7. WHO | Middle East respiratory syndrome coronavirus (MERS-CoV) - update 20 September 2013 [Internet]. WHO. [cited 2015 Sep 11]. Available from: http://www.who.int/csr/don/2013_09_20/en/

8. Abroug F, Slim A, Ouanes-Besbes L, Hadj Kacem M-A, Dachraoui F, Ouanes I, et al. Family cluster of Middle East respiratory syndrome coronavirus infections, Tunisia, 2013. Emerg Infect Dis. 2014 Sep;20(9):1527–30.

9. Premila Devi J, Noraini W, Norhayati R, Chee Kheong C, Badrul AS, Zainah S, et al. Laboratory-confirmed case of Middle East respiratory syndrome coronavirus (MERS-CoV) infection in Malaysia: preparedness and response, April 2014. Euro Surveill Bull Eur Sur Mal Transm Eur Commun Dis Bull. 2014;19(18).

10. First Confirmed Cases of Middle East Respiratory Syndrome Coronavirus (MERS-CoV) Infection in the United States, Updated Information on the Epidemiology of MERS-CoV Infection, and Guidance for the Public, Clinicians, and Public Health Authorities — May 2014 [Internet]. Morbidity and Mortality Weekly Report (MMWR); Available from: http://www.cdc.gov/mmwr/preview/mmwrhtml/mm6319a4.htm

11. European Centre for Disease Control. Epidemiological update: First imported case of MERS-CoV in Greece [Internet]. 2014. Available from: http://ecdc.europa.eu/en/press/news/_layouts/forms/News_DispForm.aspx?List=8db7286c-fe2d-476c-9133-18ff4cb1b568&ID=989

12. WHO | Middle East respiratory syndrome coronavirus (MERS-CoV) – update 1 May 2014 [Internet]. WHO. [cited 2015 Sep 11]. Available from: http://www.who.int/csr/don/2014_05_01_mers/en/

13. Breakwell L, Pringle K, Chea N, Allen D, Allen S, Richards S, et al. Lack of Transmission among Close Contacts of Patient with Case of Middle East Respiratory Syndrome Imported into the United States, 2014. Emerg Infect Dis. 2015 Jul;21(7):1128–34.

14. Centers for Disease Control and Prevention. MERS in the U.S. [Internet]. Available from: http://www.cdc.gov/coronavirus/mers/us.html

15. Middle East Respiratory Syndrome (MERS), CDC [Internet]. Available from: http://www.cdc.gov/coronavirus/mers/us.html

16. Mollers M, Jonges M, Pas SD, van der Eijk AA, Dirksen K, Jansen C, et al. Follow-up of Contacts of Middle East Respiratory Syndrome Coronavirus–Infected Returning Travelers, the Netherlands, 2014. Emerg Infect Dis. 2015 Sep;21(9):1667–9.

17. Santé-Algérie. Portail d'Information, de Documentation et de Communication - Dossier Coronavirus [Internet]. 2014. Available from: http://www.sante.dz/communique_coronavirus.pdf

18. WHO | Middle East respiratory syndrome coronavirus (MERS-CoV) – update 31 May 2014 [Internet]. WHO. [cited 2015 Sep 11]. Available from: http://www.who.int/csr/don/2014_06_14_mers/en/

19. WHO | Middle East respiratory syndrome coronavirus (MERS-CoV) – Austria [Internet]. WHO. [cited 2015 Sep 11]. Available from: http://www.who.int/csr/don/02-october-2014-mers-austria/en/

20. WHO | Middle East respiratory syndrome coronavirus (MERS-CoV) – Turkey [Internet]. WHO. [cited 2015 Sep 11]. Available from: http://www.who.int/csr/don/24-october-2014-mers/en/

21. WHO | Middle East respiratory syndrome coronavirus (MERS-CoV) – The Philippines 12 February 2015 [Internet]. WHO. [cited 2015 Sep 11]. Available from: http://www.who.int/csr/don/13-february-2015-mers/en/

22. WHO | Middle East respiratory syndrome coronavirus (MERS-CoV) – Germany [Internet]. WHO. [cited 2015 Nov 5]. Available from: http://www.who.int/csr/don/9-march-2015-mers-germany/en/

23. Middle East respiratory syndrome coronavirus (MERS-CoV): Germany reports third case [Internet]. 2015 [cited 2015 Nov 5]. Available from: http://www.euro.who.int/en/countries/germany/news/news/2015/03/middle-east-respiratory-syndrome-coronavirus-mers-cov-germany-reports-third-case

24. Choi JW, Kim KH, Cho YM, Kim SH. Current epidemiological situation of Middle East respiratory syndrome coronavirus clusters and implications for public health response in South Korea. J Korean Med Assoc. 2015;58(6):487.

25. Park H, Lee E, Ryu Y, Kim Y, Kim H, Lee H, et al. Epidemiological investigation of MERS-CoV spread in a single hospital in South Korea, May to June 2015*. Eurosurveillance. 2015 Jun 25;20(25):21169.

26. Wu J, Yi L, Zou L, Zhong H, Liang L, Song T, et al. Imported case of MERS-CoV infection identified in China, May 2015: detection and lesson learned. Euro Surveill Bull Eur Sur Mal Transm Eur Commun Dis Bull. 2015;20(24).

27. WHO | Middle East Respiratory Syndrome coronavirus (MERS-CoV) – Thailand [Internet]. WHO. [cited 2015 Sep 11]. Available from: http://www.who.int/csr/don/20-june-2015-mers-thailand/en/

28. WHO | Middle East respiratory syndrome coronavirus (MERS-CoV) – The Philippines 8 July 2015 [Internet]. WHO. [cited 2015 Sep 11]. Available from: http://www.who.int/csr/don/08-july-2015-mers-philippines/en/

29. WHO | Middle East respiratory syndrome coronavirus (MERS-CoV) – The Philippines (update) 10 July 2015 [Internet]. WHO. [cited 2015 Sep 11]. Available from: http://www.who.int/csr/don/10-july-2015-mers-philippines/en/
